# Insights into Aquatic Safety and Environmental Risks of Doped LLZO Solid-State Electrolytes

**DOI:** 10.1021/acsomega.5c08890

**Published:** 2026-02-03

**Authors:** Raphael Martinez Garcia, Marlon Muniz da Silva, Gabriela Helena da Silva, Aline Maria Zigiotto de Medeiros, Joice Janeri Gomes, Diego Stéfani Teodoro Martinez, Mathias Strauss

**Affiliations:** † Brazilian Nanotechnology National Laboratory (LNNano), 215006 Brazilian Center for Research in Energy and Materials (CNPEM), Street Giuseppe Maximo Scolfaro 10000, Campinas, São Paulo 13083-100, Brazil; ‡ School of Chemical Engineering (FEQ), University of Campinas (UNICAMP), Avenue Albert Einstein 500, Campinas, São Paulo 13083-852, Brazil; § Brazilian Synchrotron Light Laboratory (LNLS), Brazilian Center for Research in Energy and Materials (CNPEM), Street Giuseppe Maximo Scolfaro 10000, Campinas, São Paulo 13083-100, Brazil; ∥ Institute of Chemistry (IQ), University of Campinas (UNICAMP), Street Monteiro Lobato 270, Campinas, São Paulo 13083-970, Brazil; ⊥ School of Technology (FT), University of Campinas (UNICAMP), Street Paschoal Marmo 1888, Limeira, São Paulo 13484-332, Brazil

## Abstract

Solid-state batteries are considered the next advancement in lithium-ion battery technology, offering enhanced energy density and safety. However, the toxicity and environmental risks associated with the production and disposal of these materials remain insufficiently explored. This study investigates the aquatic toxicity and environmental concerns of Al- and Ta-doped garnet Li_7_La_3_Zr_2_O_12_ (LLZO), a promising solid-state electrolyte. Comprehensive material characterization was performed to evaluate the interactions of milled LLZO with aqueous environments, and potential toxicological effects of LLZO were assessed through an acute fish embryotoxicity (FET) assay using zebrafish embryos. Lithium and aluminum release were detected; however, these alterations were insufficient to induce acute toxicological effects in zebrafish embryos, even in the absence of the chorion barrier. Despite these findings suggesting the potential of doped LLZO as a less harmful alternative to conventional organic electrolytes, further studies addressing chronic exposure, species-specific responses, and broader ecological impacts are essential before LLZO can be considered environmentally safe.

## Graphical abstract

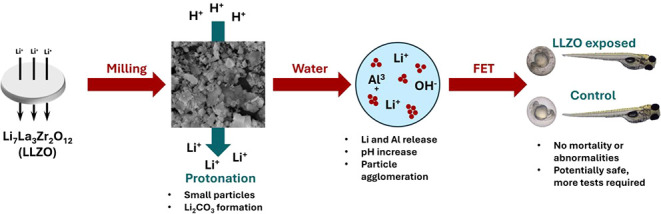

## 1 Introduction

The increasing demand for safe, inexpensive, and efficient sustainable energy sources and storage has led to significant development on rechargeable lithium-ion batteries, which are widely used in power storage systems and portable electronic devices. Recently, solid-state electrolytes (SSEs) have garnered the interest of the scientific community as a promising next step in lithium-ion battery design.^1^ By replacing conventional organic liquid electrolytes, which are highly flammable, SSEs might lead to safer battery designs. Additionally, unlike liquid electrolytes, many SSEs are compatible with metallic lithium anodes, which allow for higher energy density and mechanical stability compared to traditional graphite-based anodes.^2–8^ Among the various SSEs, garnet-like cubic Li_7_La_3_Zr_2_O_12_ (LLZO) ceramics are well-established in the literature as one of the most promising materials due to their high ionic conductivity (between 10^–4^ and 10^–3^ S cm^–1^), improved electrochemical stability, including against metallic lithium, and high mechanical strength.^9–12^

Currently, large-scale production of LLZO has not yet been fully established. First reported in 2007,^10^ cubic LLZO is a relatively new material, and the transition from laboratory-scale to industrial-scale production has only recently begun. The solid-state synthesis route offers an easily scalable approach, making it a promising method for industrial manufacturing of LLZO.^6,13^ Traditionally, this method begins by milling the precursors into a fine and homogeneous powder, which is then calcined at high temperatures to form cubic LLZO. The material is milled again to reduce particle size, formed into the desired SSE shape, and then sintered to reduce porosity and achieve the desired electrochemical and mechanical properties.^14–17^ Since cubic LLZO is thermodynamically unstable and tends to transition to the tetragonal phase, which is undesirable due to lower ionic conductivity, it is typically doped with metallic atoms such as Al, Ta, and Nb. These dopants help stabilize the cubic phase and often enhance overall performance.^4,18–21^

While SSEs are generally considered safe due to their nonflammability,^22^ recent studies indicate that their status as “safe materials” needs to be reconsidered. During operation, interfacial reactions between many SSEs and electrodes can lead to thermal runaway and the release of hazardous gases. In addition, several SSE materials release dangerous compounds upon exposure to ambient moisture, including sulfides (H_2_S and CO gases), chlorides (corrosive HCl), and hydrides (flammable H_2_ gas).^23,24^ Consequently, improper disposal of these materials during manufacturing or after use may result in contamination of aquatic and terrestrial ecosystems. Oxide-based SSEs, including LLZO and other promising materials such as Li_1+*x*_Al_*x*_Ge_2–*x*_(PO_4_)_3_ (LAGP) and Li_1+*x*_Al_*x*_Ge_2–*x*_(PO_4_)_3_ (LATP), are considered the most chemically stable SSEs and are therefore speculated to be safer. However, despite their chemical stability, data on the toxicity and other aspects of safety for these materials remain limited in the literature.

LLZO is no exception, as there is limited knowledge regarding the environmental impacts associated with its production and disposal.^6^ So far, life cycle assessments indicate that the most environmental burdens arise from high energy demands and the use of lanthanum-based precursors.^2,12,25^ However, no studies have yet addressed LLZO toxicity or its effects on the environment. Accordingly, the assessment of the ecotoxicological effects of LLZO-related materials is essential to substantiate the environmental safety of LLZO across its life cycle. Regarding potential environmental impacts associated with synthesis via the solid-state route, the powder generated after the milling of the calcined material is considered critical. This milled product consists of small particles, typically within the nanometric and/or submicrometric range, resulting in high surface area, increased reactivity, and greater potential for environmental translocation, all of which may intensify toxicological effects. Nanoparticles are particularly concerning, as their high mobility allows them to cross protective barriers more easily, accumulate within animal organs and cause damage.^26–28^

Water effluents can dissolve and transport various inorganic and organic chemical compounds, making aquatic environments particularly vulnerable to multiple contamination pathways.^29^ In this context, the acute fish embryo toxicity (FET) test is a well-established assay for evaluating the toxicity of chemicals on fish embryonic development.^27,28,30–33^ In typical FET assays, embryotoxic and teratogenic effects resulting from exposure are assessed during the early developmental stages of fertilized zebrafish (*Danio rerio*) eggs, providing valuable insights into material toxicity.^33^ A notable variation of this assay involves the removal of the chorion barrier, a protective membrane surrounding the embryo. This modification allows direct contact between the embryo and the material, potentially enhancing the relevance of observed effects to other vertebrate models.^32^

This study aims to provide initial insights into the environmental impacts of Ta- and Al-doped LLZO. Advanced material characterization confirmed the formation of Li_2_CO_3_ resulting from LLZO protonation during milling, which leads to high lithium availability in the tested environment. Additional aspects of LLZO behavior in aquatic environments were investigated, including the leaching of other constituents, pH increase, and particle agglomeration. Moreover, an acute zebrafish FET assay was conducted to identify potential major toxicological effects associated with LLZO exposure, including conditions where the chorion barrier was removed. Finally, this work discusses multiple factors that may influence LLZO aquatic toxicity and proposes an initial study for environmental concerns around this promising material, aiming to support future safe-by-design technological applications of LLZO.

## 2 Methods

### 2.1 Chemicals

Li_2_CO_3_ (CAS 554-13-2, Alfa Aesar, 99.998%), La_2_O_3_ (CAS 1312-81-8, Alfa Aesar, 99.9%), ZrO_2_ (CAS 1314-23-4, Alfa Aesar, 99.7%), Ta_2_O_5_ (CAS 1314-61-0, Alfa Aesar, 99.993%), Al_2_O_3_ (CAS 1344-28-1, Alfa Aesar, 99.5%), Isopropyl alcohol (CAS 67-63-0, Merck, 99.7%).

### 2.2 Solid-State Synthesis of LLZO

The synthesis process was followed as described in the Supporting Information. Precursors were weighed to produce LLZO following the stoichiometries of Li_6,1_La_3_Zr_2_Al_0,3_O_12_ (LLZAO) and Li_6,6_La_3_Zr_1,6_Ta_0,4_O_12_ (LLZTO), with an additional 15% wt. of the Li precursor being added to compensate for lithium losses during the synthesis process. The precursors were milled, pelletized and then calcined, transforming into LLZAO and LLZTO. The LLZAO and LLZTO samples were crushed and subsequently milled in isopropanol at 1000 rpm for 6 h.

### 2.3 Fish Embryo Toxicity Assay (FET)

*D. rerio* embryos (wild-type) were obtained from the Brazilian Nanotechnology National Laboratory (LNNano–CNPEM, Campinas, Brazil). The acute toxicity of the materials (milled LLZAO and LLZTO) was determined according to OECD TG 236.^33,34^ One hour after a natural mating of wild-type adults, the eggs were collected and washed in reconstituted water (RW). RW was prepared as moderately hard water following USEPA (2002).^35^ The solution consists of NaHCO_3_ (96 mg L^–1^), MgSO_4_ (60 mg L^–1^), KCl (4 mg L^–1^) and CaSO_4_·2H_2_O (60 mg L^–1^) dissolved in ultrapure water (UPW, Milli-Q type 1) and has a pH of 7.0 ± 0.5. Viable eggs were selected under a stereo microscope. Zebrafish eggs with less than 4 h post fertilization (hpf) and 24 hpf dechorionated embryos were transferred to 24-well polystyrene plates with 2 mL of the test solution and 1 embryo per well, 20 embryos per treatment. For the chorion removal, the eggs were dechorionated mechanically with forceps (Dumont no.5) according to the reported procedure.^35^ The tested concentrations were 1.0, 10, and 100 mg L^–1^ of milled LLZAO and LLZTO, which were previously dispersed by sonication for 30 min in RW. Moreover, a negative control group (only RW) was also performed. The test plates were then placed in an incubator at 28.0 ± 1.0 °C under a 10/14 h dark/light regime. The embryos were analyzed under a stereomicroscope (Zeiss), and all developmental alterations were documented at 24, 48, 72, and 96 hpf. At the end of the exposure period (96 hpf), the live larvae were photographed and then measured using ImageJ software. The measurements of the larvae’s total length were performed from the beginning of the eye to the tip of the tail. FET tests with a minimum mortality rate of 30% in the positive control (4 mg L^–1^ 3,4-dichloroaniline) and a maximum effective rate of 10% in the negative control at 96 hpf were classified as valid. FET tests were run in triplicates. Statistical analysis was performed using OriginPro 2022b (OriginLab). Additional details are provided in the Supporting Information.

### 2.4 Environmental Exposure

Milled LLZAO and LLZTO samples were dispersed in UPW and RW at concentrations of 100 mg L^–1^ through sonication for 10 min (unless stated otherwise), left undisturbed for different periods (total exposure times of 15 min, 1 h, 6 h, 24 h and 96 h, including the sonication period), and subsequently separated from the aqueous medium by centrifugation, followed by drying. The remaining powders and supernatants were then characterized using multiple analytical techniques. For the ICP-OES measurements of the aqueous media, powder separation was performed through four consecutive centrifugations at 11000 rpm for 5 min, aiming to remove as much powder as possible.

### 2.5 Instrumentation

X-ray diffraction (XRD) patterns of the powder samples before milling, after milling and after immersion in RW for 96 h (30 min of initial sonication) were recorded at 2θ ranges of 15–90° on a Bruker D8 Advance Eco diffractometer with Cu Kα_1_ radiation (λ = 1.5406 Å) at 40 kV. Reference patterns^36–39^ were extracted from CIF files within the Crystallography Open Database^40^ through the software QualX 2.0.^41^ Rietveld refinements were conducted using TOPAS (Bruker, V5) software. Raman spectroscopy of the milled samples, before and after immersion in UPW and RW for 96 h (30 min of initial sonication), was conducted using a Horiba XploRA PLUS confocal Raman microscope. The laser excitation wavelength was 532 nm, and the spectra were collected over a range of 50–1500 cm^–1^, with 10 accumulations of 10 s each. Data were collected randomly and punctually across the powder samples, which were placed on microscope glass slides. Scanning electron microscopy (SEM) images of the milled powders were obtained using a Thermo Fisher Quanta 650 FED microscope, operating at 10 kV with a 50 pA current in secondary electron (SE) mode. Energy-dispersive X-ray spectroscopy (EDS) was performed simultaneously with SEM. The specific surface areas of the milled powders were determined using the BET method applied to N_2_ adsorption–desorption measurements on a Micromeritics ASAP 2020 analyzer.

Dynamic light scattering (DLS) measurements of the milled powders dispersed in UPW were performed after different periods of ultrasonication using a bath sonicator, employing a Malvern Zetasizer Ultra analyzer (Malvern, UK). After 90 min of sonication, aliquots of the dispersions were transferred into RW and analyzed by DLS. Zeta potential measurements of the milled powders in UPW were conducted concurrently with DLS using the same instrument. Li, La, Zr, Al, and Ta concentrations in UPW and RW before and after exposure to 100 mg L^–1^ of milled LLZAO and LLZTO for different conditions (15 min, 1, 6, 24, and 96 h with 10 min of sonication; 96 h without sonication) were measured by ICP-OES using a PerkinElmer Optima 8000 analyzer in replicates of at least three. The pH of these same samples was measured using pH strips (Macherey-Nagel 92122, pH 6.0–10.0, precision ± 0.3 or Kasvi K36-014f, pH 0–14, precision ± 1). Statistical analysis of the ICP-OES and pH measurements was performed using OriginPro 2025 (OriginLab). Na, Mg, K, and Ca concentrations from redispersions, in UPW, of the milled LLZAO and LLZTO powders immediately after recovery (via centrifugation) from UPW and RW (96 h of exposure, 30 min of sonication) were measured in duplicates by ICP-OES using the same instrument. To stabilize the dissolved metal species, 2 vol % of concentrated HNO_3_ was added to the solutions prior to ICP-OES measurements.

## 3 Results and Discussion

### 3.1 Material Characterization

The milled LLZAO and LLZTO particles presented similar size and morphology, both irregular in shape and highly polydisperse, as expected from milling (Figure 1A). Overall, particle size was predominantly at the submicron range, although several nanometric and some micrometric particles were observed. The particle size parameters (size, polydispersity, and surface area) are provided in the Supporting Information (Table S1). The XRD patterns of both materials before milling (Figure 1B) confirm that addition of either Al or Ta culminates in the formation of cubic LLZO, although traces of secondary phases were detected in both cases (see Supporting Information, subsection S2.1). The absence of peaks attributed to Li_2_CO_3_ in these samples indicates that the excess of this precursor was decomposed during calcination, with the Li excess either volatilized or incorporated into the crystal structure.^42,43^

**Figure 1:**
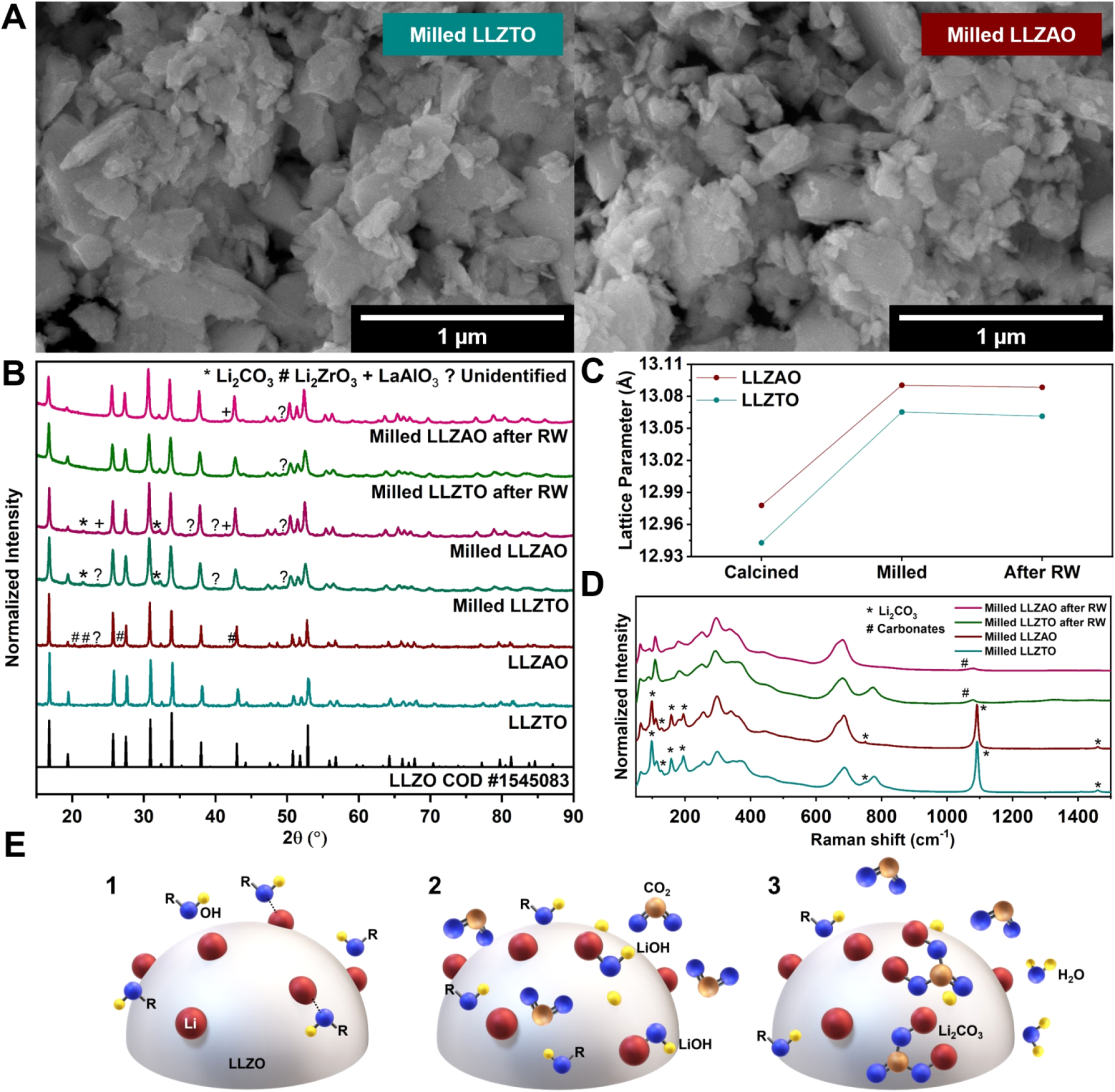
(A) SEM images of the milled LLZTO and LLZAO samples. (B) XRD diffractograms of the LLZTO and LLZAO samples before milling, after milling, and after exposure to RW for 96 h (30 min of sonication). Secondary phases are shown in detail in the Supporting Information (Figure S1A). (C) The lattice parameters of the LLZTO and LLZAO samples were calculated from Rietveld refinement of the XRD data. (D) Raman spectra of the milled LLZTO and LLZAO samples before and after exposure to RW for 96 h (30 min of sonication). (E) Schematic representation of the protonation of LLZO particles during the milling process: (1) initial interaction of OH radicals with Li atoms at the surface; (2) formation of LiOH on the surface followed by exposure to CO_2_; and (3) formation of Li_2_CO_3_ on the LLZO surface accompanied by the release of H_2_O.

Major changes in the XRD diffractograms of both materials were observed after milling (Figure 1B). The peak broadening is attributed to a reduction in crystallite size caused by the milling process. Moreover, the lattice parameter, as determined from Rietveld refinement, increased significantly, strongly suggesting protonation during this process. Protonation in LLZO refers to the partial substitution of Li^+^ ions in the crystal structure by H^+^ cations, which can occur during milling with protic solvents such as water, ethanol, or isopropanol,^44^ as well as during prolonged exposure to humid environments, including atmospheric air or water.^45,46^ The main evidence for protonation is the lattice parameter increase (Figure 1C), which results from the replacement of stronger Li–O bonds with weaker H–O bonds.^45^ Li^+^ ions removed from LLZO through protonation react with atmospheric moisture to form LiOH, which subsequently reacts with CO_2_ to form Li_2_CO_3_, resulting in the formation of a LiOH–Li_2_CO_3_ layer on the particle surfaces (Figure 1E).^12,19,45–47^ This process can be described by the reaction pathways like the one presented in eqs 1 and 2, which shows the case of water-induced protonation.^48^ Consistent with this mechanism, both materials exhibited a minor diffraction peak corresponding to crystalline Li_2_CO_3_ in their XRD diffractograms after milling.

1 $$Li_{7}La_{3}Zr_{2}O_{7}+xH_{2}O\to Li_{7-x}H_{x}La_{3}Zr_{2}O_{7}+xLiOH$$

2 $$2LiOH+CO_{2}\to Li_{2}CO_{3}+H_{2}O$$

However, the LiOH–Li_2_CO_3_ layer may be partially amorphous and is often inadequately characterized by XRD.^49–52^ Raman spectroscopy is a more suitable technique for characterizing this layer and was applied to the milled LLZAO and LLZTO samples before and after exposure to RW (Figure 1D). The presence of Li_2_CO_3_ in both materials prior to RW exposure is evident from spectral signatures at 97, 157, and 196 cm^–1^, along with a sharp, high-intensity peak at 1090 cm^–1^ characteristic of carbonates.^47^ In contrast, peaks corresponding to LiOH (around 330 cm^–1^ and 620 cm^–1^)^53,54^ and LiOH·H_2_O (around 144, 212, 245, 518, and 840 cm^–1^)^55,56^ were not observed, indicating complete conversion of hydroxide species in the LiOH–Li_2_CO_3_ layer into Li_2_CO_3_. The remaining peaks are characteristic of LLZO, with differences between the LLZAO and LLZTO spectra attributed to their respective dopants.^20,57–60^

After exposure to RW, neither LLZAO nor LLZTO exhibited a further increase in lattice parameters (Figure 1C), suggesting that no additional LLZO protonation occurred. Based on ICP-OES measurements, we estimated that protonation resulted in a Li loss of 60 ± 3% for LLZTO and 61 ± 3% for LLZAO (see Supporting Information, subsection S2.2). Moreover, crystalline Li_2_CO_3_ was no longer detected in the XRD diffractograms (Figure 1B), and most of the corresponding Raman signatures had also disappeared (Figure 1D). The only exception was the characteristic carbonate peak at 1090 cm^–1^, which persisted with greatly reduced intensity. Since Li_2_CO_3_ is relatively soluble in water,^61^ this suggests that the surface layer was removed by dissolution. The remaining carbonate signal indicates the presence of residual carbonate phases, which may not correspond exclusively to Li_2_CO_3_, as other metallic species in RW interacted with the material after sample removal and could have formed secondary carbonates (see Supporting Information, subsection S2.1).

### 3.2 Material Behavior under Aqueous Media

The release of Li upon immersion of milled LLZO in RW was confirmed by ICP-OES analysis (Figure 2A), consistent with the dissolution of Li_2_CO_3_. After exposure of LLZO at 100 mg L^–1^, Li concentration in RW increased from 26.1 ± 0.1 μM to 564 ± 35 μM on average, with no significant difference between LLZTO and LLZAO. Additionally, a substantial increase in Al concentration in RW was detected after exposure to LLZAO, rising from 1.0 ± 0.2 μM to 3.3 ± 1.1 μM on average. No statistically significant increases in Ta, La, or Zr concentrations were observed in UPW or RW after exposure to either LLZAO or LLZTO. Schneider et al.^48^ reported that, upon immersion of Al- and Ta-doped LLZO in water, elements such as La, Zr, Ta and structurally incorporated Al remain stable, while nonincorporated (intergranular) Al is susceptible to leaching. In agreement with this, SEM-EDS mapping of the LLZAO sample (see Supporting Information subsection S2.1) revealed Al-rich domains, which are a likely source of Al release in water.

**Figure 2:**
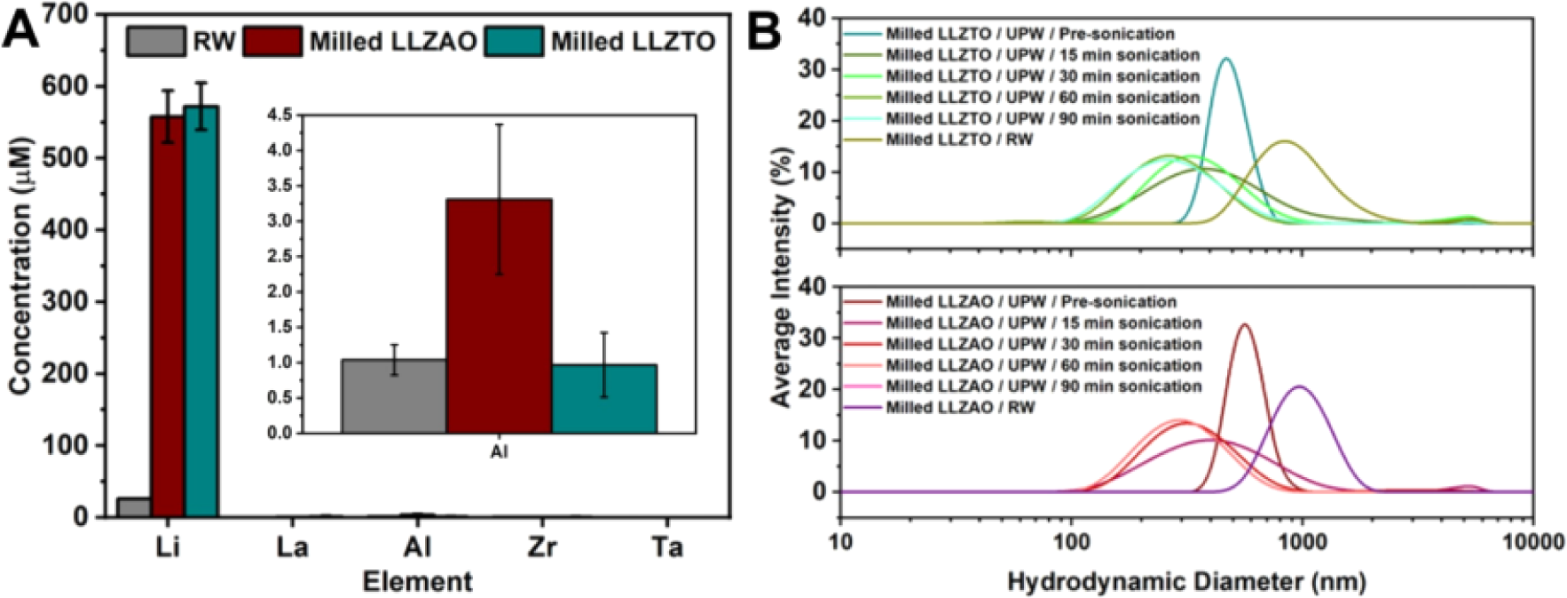
(A) Concentrations of Li, La, Zr, Al, and Ta obtained from ICP-OES measurements of RW and the supernatants of 100 mg L^–1^ milled LLZTO and LLZAO dispersions in RW, with an inset highlighting the Al concentration. Average values and standard deviations (error bars) were calculated considering all exposure periods and sonication conditions (see Supporting Information, subsection S2.2). (B) Hydrodynamic diameter distribution of milled LLZTO and LLZAO dispersions in UPW (with varying sonication duration) and in RW (without sonication).

The measured Li concentration in RW may be overestimated due to signal interference from the metallic species added to RW (Na, K, Mg, and Ca). Furthermore, in RW, no correlation was observed between Li concentration and either exposure time or sonication time. In contrast, in UPW, Li concentration was directly proportional to sonication time and, possibly, to exposure time, likely due to particle deagglomeration and additional protonation during the later stages of exposure. A detailed discussion of the ICP-OES results is provided in the (Supporting Information subsection S2.2).

The hydrodynamic size of LLZAO and LLZTO particles in water was monitored using DLS measurements (Figure 2B). In UPW, both samples exhibited hydrodynamic diameter peaks around 500 nm. Upon sonication, the size distributions gradually shifted toward smaller values, with peaks near 250 nm observed after 60 min or longer. The particles were initially agglomerated, likely due to the drying step following milling. Sonication enabled deagglomeration, facilitated by electrostatic repulsion between particles arising from their electrical double layer, as indicated by a zeta potential of approximately −40 mV (Table S1, Supporting Information). When the deagglomerated particles were introduced into RW, the hydrodynamic diameters increased immediately, indicating significant agglomeration. This behavior is attributed to the ionic species present in RW, which likely disrupted the electrical double layer and reduced electrostatic repulsion forces, thereby promoting particle aggregation. Consequently, the hydrodynamic diameters in RW remained consistently above 400 nm, suggesting the absence of nanometric or similarly small agglomerates.

Moreover, the dispersion of LLZO in aqueous media typically leads to an increase in pH because direct protonation produces LiOH according to eq 1. LiOH is a strong base, so concentrated LLZO dispersions often exhibit pH values in the range of 11–13.^62–65^ At ambient temperature and in exposure intervals up to multiple days, LLZO generally loses up to 50–60% of its Li content in water.^45,46,48^ Hence, considering eq 1 and assuming complete LiOH dissociation, the expected pH value for nominal LLZO dispersions in water at a concentration of 100 mg L^–1^ would be approximately 10.6–10.7. However, the pH of milled LLZAO and LLZTO dispersions measured in RW was 8.0 ± 0.3 during the first 24 h of exposure and 7.3 ± 0.3 at 96 h, with no differences between LLZAO and LLZTO, nor correlation with sonication time. The observed pH increase was likely lower than expected because the samples were partially protonated during milling, which limited further protonation and consequently LiOH formation. In addition, the LiOH produced from LLZO protonation during milling was fully converted to Li_2_CO_3_, a considerably weaker base than LiOH, prior to exposure to RW. Moreover, because the pH remained relatively close to neutral, the equilibrium between carbonate species and atmospheric CO_2_ possibly acted to buffer additional pH variations and could have contributed to the observed pH decrease between the first 24 h and 96 h of exposure.^66,67^ Further details on the pH measurements are provided in the (Supporting Information subsection S2.2).

### 3.3 Milled LLZO Zebrafish FET Assay

Across all tested concentrations, the total length of larvae remained within the lower and upper limits of the control group (3.99 ± 0.13 mm with chorion, 3.88 ± 0.17 mm without chorion, intervals defined using standard deviation), regardless of the presence or absence of the chorion barrier (Figure 3A,B). Detailed statistical analysis is provided at (Supporting Information subsection S2.3). Furthermore, no mortalities or malformations were observed under any exposure condition, even at the highest particle concentrations (Figure 3D,E) and in the absence of the chorion barrier, despite the visible interaction between LLZO particles and the chorion barrier (Figure 3C). These results indicate, with high confidence, that exposure to up to 100 mg L^–1^ of milled Al- or Ta-doped LLZO does not induce lethality or malformations during the first 96 h of zebrafish embryonic development.

**Figure 3:**
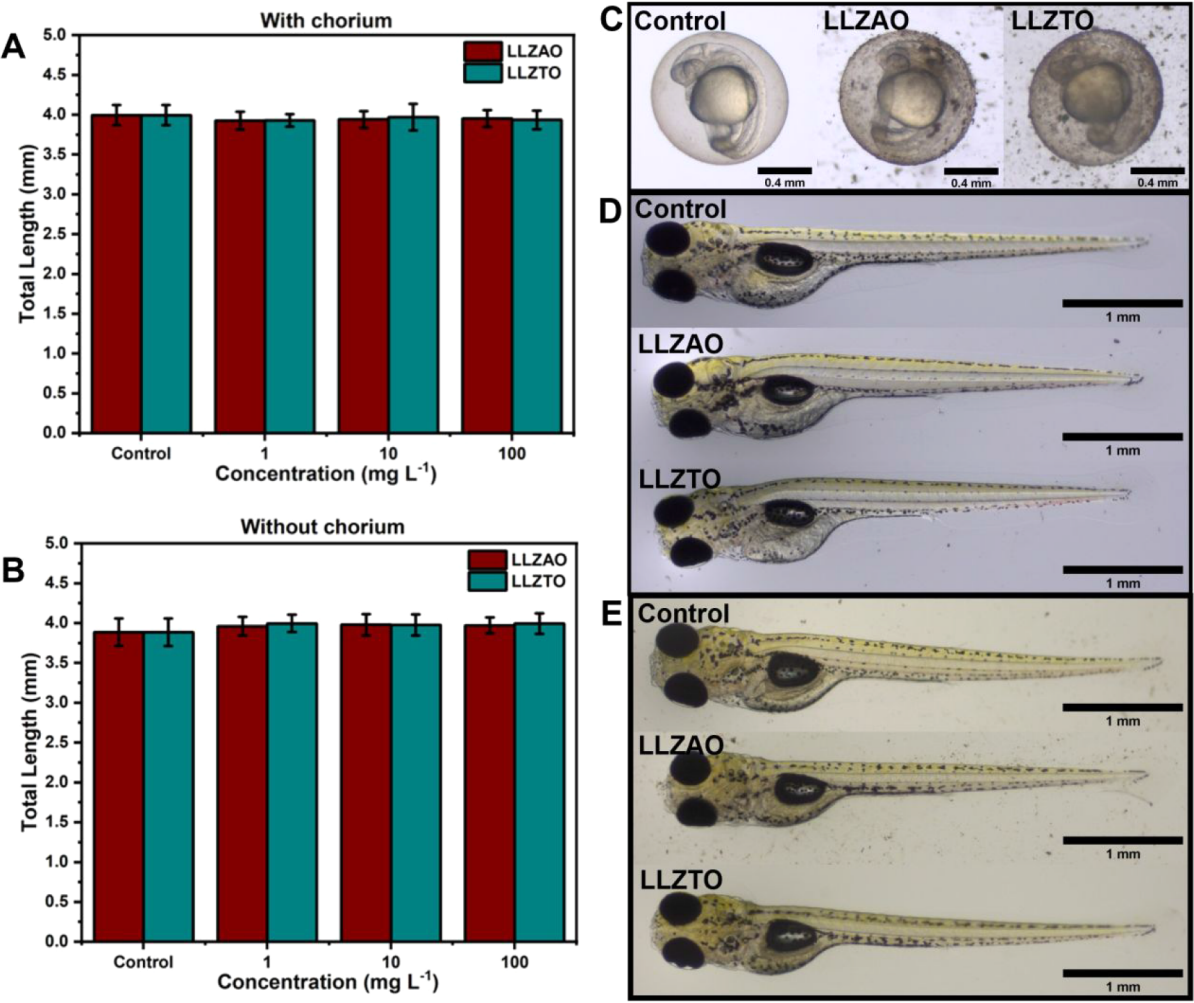
(A) Total length of the embryo 96 hpf in the assay with chorion. (B) Total length of the embryo 96 hpf in the assay without chorion. Error bars indicate standard deviation. (C) Embryo with chorion 24 hpf. (D) Final embryo 96 hpf with chorion. (E) Final embryo 96 hpf without chorion.

Li exposure can cause lethality or developmental abnormalities in zebrafish embryos, typically appearing after 48 hpf, with skeletal deformations in the tail being among the most common manifestations.^31,68,69^ Other morphological, physiological, hatching and behavioral effects have also been reported. However, toxic thresholds vary widely depending on water parameters such as Na and K concentration, hardness, pH, and dissolved organic carbon (Table 1).^70–73^ In this context, the Li concentrations measured in RW after exposure to milled LLZAO and LLZTO (564 μM ± 35 μM) were below the levels reported to induce abnormalities at 96 hpf in most studies, consistent with the absence of observable effects.

**Table 1:** Reported Toxicological Effects of Acute Li Exposure on Zebrafish Embryos

Refs	Abnormalities	Start^a^	End^a^	Measurement^a^	Mortality parameters^a^	Abnormality parameters^a^
 ^68^	Somite defects, decreased heartbeat rate, no blood circulation, delayed hatching, tail kink, absence of side profile, decreased swimming activity	<2 hpf	24–48 hpf	24–48 hpf	NOEC 58.9 mM, LOEC 236 mM (100% mortality)	NOEC 59 mM
			72 hpf	72 hpf		EC50 38.5 mM
			144 hpf	144 hpf	NOEC 14.7 mM, LOEC 58.9 mM, LC50 53.4 mM	EC50 10.7 mM
^69^	Dorsal curvature, pericardial edema, decreased heartbeat rate, decreased swimming activity and velocity, variations in gene expression	4 hpf	48 hpf	48 hpf	NOEC 20 mM	NOEC 20 mM
		48 hpf	72 hpf	72 hpf	LC50 208 μM	EC50 232 μM for morphological abnormalities, LOEC < 50 μM for physiological abnormalities
				144 hpf	LC50 179 μM	EC50 151 μM for morphological abnormalities, LOEC 150 μM for swimming activity
^74^	Eye defects	24 hpf	48–240 hpf	48–240 hpf	NOEC 150 mM, LOEC 300 mM, 100% mortality at 450 mM	LOEC 150 mM for eye defects. Failed eye development at 300 mM
^75^	Delayed hatching, decrease in locomotion and exploratory patterns	Unspecified	72 hpf	240 hpf	NOEC 5 mM	NOEC 5 mM for morphological abnormalities, LOEC 5 mM for hatching delay, LOEC 500 μM behavioral abnormalities
^76^	Increase in pigmentation	24 hpf	144 hpf	144 hpf	No mortality data was reported	LOEC 50 mM
^77^	No abnormality data was reported	Unspecified	96 hpf	96 hpf	LC50 2.60 mM	No abnormality data was reported
^78^	Embryo coagulation, no movement, no blood circulation, heart edema, small eyes, scoliosis, short tail, underdeveloped fins	4 cell stage	48 hpf	48 hpf	No mortality data was reported	NOEC 160 mM
			144 hpf	144 hpf		NOEC 30 mM, LOEC 40 mM

a HPF = hours post fertilization; NOEC = no observed effect concentration; LOEC = lowest observed effect concentration; EC50 = median effective concentration; LC50 = median lethal concentration.

Regarding the other LLZO constituents, potential toxicological effects from La, Zr, and Ta were absent, likely because these elements were not released upon immersion in RW. In contrast, Al release from LLZAO (Figure 2A, inset) could potentially cause severe effects, including pericardial edema, reduced heartbeat, and brain damage.^79–82^ At 96 hpf, significant mortality and morphological abnormalities have been observed at concentrations as low as 5 μM,^80,81^ though not necessarily at 2.5 μM.^81^ Therefore, the measured Al concentration in RW after LLZAO exposure (3.3 ± 1.1 μM) was likely below the threshold needed to induce observable morphological or lethal effects.

Potential toxicological effects associated with small particle size were likely mitigated by agglomeration, which increased the effective particle size enough to prevent embryo uptake, even during direct contact following removal of the chorion.^27,28,85^ Furthermore, the pH increase resulting from LLZO exposure could potentially affect zebrafish embryos, as alkaline conditions can inhibit ammonia excretion, disrupt acid–base and ionic regulation, and induce other toxicological effects.^84–87^ However, such effects are typically observed only at pH values above 10,^83,88^ indicating that the pH rise induced by milled LLZO was insufficient to cause significant toxicity.

Overall, the measured concentrations of Li, Al, and other LLZO constituents, combined with the moderate pH increase and particle agglomeration, are consistent with the absence of mortality, malformations, or developmental delays observed in the acute zebrafish FET assay, indicating minimal toxicological impact under the tested conditions.

### 3.4 LLZO Aquatic Toxicology: Advantages, Concerns, and Aggravating Factors

Conventional Li-ion electrolytes contain organic solvents and LiPF_6_, which can reduce hatching, impair swim bladder development, and induce multiple morphological abnormalities in zebrafish embryos.^89–92^ Furthermore, some components can be decomposed into highly toxic products, such as LiPF_6_, which may degrade into HF.^93,94^ In this context, LLZO-based electrolytes appear to offer improved toxicity profiles and enhanced environmental safety.

However, the FET assay primarily detects lethality and major morphological abnormalities, while more subtle effects, including hatching and behavioral changes, were not assessed and may still occur. Li exposure is known to induce behavioral abnormalities in zebrafish embryos by inhibiting glycogen synthase kinase 3 (GSK3), a key regulator of the Wnt/β-catenin signaling pathway, which controls cellular events across multiple tissues^31^ and is critical for brain development. Disruption of this pathway can lead to decreased swimming activity, lethargy, and thigmotaxis (a preference for the edges of a novel environment) at later developmental stages.^75^ Importantly, such behavioral effects can manifest at lower concentrations than those required to induce morphological abnormalities and may persist after exposure due to early malformations or neurodevelopmental damage.^69,75^

LLZO aquatic toxicity may also depend on the composition of the material, particularly the dopants. Ta appears safe, as it is not released in aqueous media, whereas Al poses a potential risk. The LLZAO produced in this study released only a small fraction of Al (8.7 ± 1.5%, see Supporting Information, subsection S2.2, of the nominal stoichiometry), preventing major toxic effects. However, in milled LLZO batches with poor Al incorporation, Al release can exceed 40% in water,^49^ potentially resulting in pronounced toxicity. Although occurring via a mechanism different from Li, Al contamination can also cause severe brain damage in developing zebrafish, leading to behavioral abnormalities.^79–81^

In this regard, LLZO aquatic toxicity may vary considerably depending on the synthesis method. While solid-state synthesis is promising for its industrial-scale production due to its simplicity, it often yields a material with relatively low compositional homogeneity and some impurities.^13^ For the synthesis of Al-doped LLZO, these factors can contribute to the formation of intergranular Al, increasing Al release into aquatic environments. In contrast, chemical routes typically produce powders with a high degree of uniformity. However, the resulting particles often exhibit morphologies and surface properties that differ substantially from those obtained by solid-state synthesis,^95^ which may influence their toxicity in multiple ways. Moreover, chemical routes frequently use hazardous chemicals with detrimental effects on the environment.^13^

Likewise, the aquatic toxicity and environmental impact of LLZO may depend on its morphology, agglomeration state, and age. Fresh LLZO powders are likely more toxic than milled or aged powders because they undergo direct protonation in water, which can increase the pH to lethal levels (>10 for zebrafish)^84,88^ within minutes.^63^ Furthermore, as discussed in the (Supporting Information subsection S2.2), particle agglomeration can influence protonation and, consequently, toxicity. This is particularly relevant for fresh LLZO, which may contain large agglomerates and aggregates due to the lack of milling, slowing both the release of Li and the increase in pH. In addition, agglomerates exhibit reduced mobility in aquatic environments and can be more easily removed through simple separation methods such as sedimentation or filtration. Sintered LLZO pellets, with low surface area, are also more resistant to protonation and Al leaching, which reduces and slows Li and Al release as well as pH increase.^48,63^ Therefore, toxicity is likely more pronounced during intermediate production stages than in the final form.

The main sources of LLZO toxicity in aquatic environments, protonation and intergranular Al release, are directly proportional to LLZO concentration. In this study, the FET assay was conducted using a range of screening concentrations (1.0, 10, and 100 mg L^–1^) to establish a preliminary dose–response relationship, as recommended by OECD guidelines.^34,96^ Although the absence of toxicity within this range suggests that LLZO has low or negligible toxicity, at least toward zebrafish embryos, detrimental environmental effects may still occur at higher concentrations, such as those found near industrial effluents. During milling, for instance, LLZO concentrations can exceed 50% wt.^44,97^ and improper disposal of material from this stage into aquatic environments could lead to local LLZO concentrations above 100 mg L^–1^ near the discharge site, along with the release of potentially toxic additives and organic solvents. Moreover, the characteristics of the receiving water body are also relevant. For example, in lotic systems such as rivers, turbulence can affect particle agglomeration,^98^ which can influence toxicity.

Several other water parameters may also modulate LLZO toxicity. The overall effects of chronic Li exposure tend to decrease under alkaline conditions,^70^ whereas toxicological effects in *Daphnia magna* neonates increase significantly in acidic waters.^71^ Furthermore, dissolved salts strongly influence Li toxicity. Sodium is known to mitigate Li toxicity in multiple species, and natural Na concentrations may even eliminate it entirely for some.^70,72^ Similarly, potassium has been shown to reduce Li toxicity in *Oncorhynchus mykiss*,^99^ while water hardness, determined by calcium and magnesium concentrations, plays an important role in acute Li toxicity, which is generally lower in moderately hard waters (≥100 mg L^–1^ CaCO_3_) than in soft waters.^70^ Dissolved organic carbon can also substantially reduce Li toxicity, and other water quality parameters that are often not measured may also be relevant to LLZO toxicity.^70^ In addition, the DLS results (Figure 2B) indicate that dissolved salts can affect LLZO particle agglomeration, which may influence its toxicity and environmental behavior.

Other water parameters that may exacerbate LLZO toxicity include temperature, as LLZO protonation in water is favored at high temperatures,^45^ and the presence of contaminants. Recent studies on long-term exposure of *Daphnia magna* to Li and microplastics showed that the combination of these emerging contaminants can reduce reproductive success by up to 93%.^100^ Moreover, acidification of water, even with weak acids such as citric acid, can promote protonation and enhance the leaching of Li, Al, La, Zr, and Ta from LLZO.^45,48^ La toxicity is well documented, with lethality and morphological, physiological, behavioral, and hatching abnormalities reported in zebrafish embryos at 10–100 μM, and toxic effects below 100 μM observed in other aquatic species.^101–104^ In contrast, Zr and Ta are generally considered low-toxicity elements for most aquatic organisms, mainly because they tend to precipitate in natural waters, reducing their bioavailability.^104–107^ Nonetheless, their aquatic toxicity remains poorly understood. Several studies have reported toxic effects of Zr on aquatic organisms despite its low solubility,^105,108,109^ whereas for Ta the evidence is more limited, but available studies suggest it can bioaccumulate in aquatic environments.^110^ In summary, LLZO toxicity may vary significantly among different aquatic environments. In particular, LLZO behavior and toxicity in saline water bodies may differ greatly from those reported here because they contain many chemical species that can interact with LLZO in unpredictable ways.

Finally, despite the seemingly encouraging outcomes of the acute FET assay, these results are not sufficient to classify LLZO as environmentally safe. The effects of Li exposure on later developmental stages, the life cycle of the material in natural environments, and the potential chronic toxicity associated with LLZO remain largely unexplored. A major concern is the potential for LLZO particles to bioaccumulate and undergo biomagnification within aquatic food webs. Filter-feeding organisms, for example, may accumulate LLZO particles, which can then be transferred to higher trophic levels through ingestion.^111^ Once ingested, LLZO is exposed to acidic digestive fluids that can leach the metallic constituents, increasing the risk of systemic toxic effects. Furthermore, toxicity assays on other organisms and environments are essential before LLZO can be considered a safe material.

## 4 Conclusions

In this study, we investigated the potential environmental risks associated with the release of Al- and Ta-doped cubic LLZO into aquatic environments. Cubic LLZO was synthesized using a solid-state route, and the effects of the material on *Danio rerio* embryos were assessed through FET assays conducted at a critical stage of solid electrolyte production, immediately after the milling process. This work represents an important contribution to the safety-by-design approach, as it evaluates LLZO safety from the early stages of production.

XRD and Raman spectroscopy revealed the formation of Li_2_CO_3_, resulting from LLZO protonation during milling. ICP-OES confirmed the release of Li and Al into water, although concentrations remained below lethal levels for zebrafish. Similarly, the moderate pH increase caused by milled LLZO was below the toxicological threshold for zebrafish. DLS measurements further showed that LLZO particles agglomerate in aqueous media, increasing their effective size and thereby reducing uptake and acute effects. FET assays with zebrafish indicated that, despite ion release, no acute adverse effects were observed at concentrations up to 100 mg L^–1^.

Overall, these results suggest that LLZO exhibit absence of acute toxicity under the tested conditions and may represent a safer alternative to conventional organic electrolytes. The findings allowed the identification of safe concentration to guide more advanced studies. Nevertheless, further research is needed to evaluate potential long-term effects, responses in other aquatic species, interactions with natural organic matter and cocontaminants, and ecological implications across diverse environments before LLZO can be considered environmentally safe.

## Supplementary Material



## Notes

Experimental procedures for the solid-state synthesis of LLZO are provided in Section S1; complementary results and discussion are presented in Section S2, (i) compositional analysis of the LLZAO and LLZTO powders (Subsection S2.1); (ii) detailed discussion of ICP-OES concentration and pH measurements (Subsection S2.2); and (iii) statistical analysis of the zebrafish FET assay (Subsection S2.3); results from characterization techniques used in Subsection S2.1 are shown in Figure S1; statistical analysis results and linear fittings discussed in Subsection S2.2 are provided in Tables S2–S7 and Figure S2; statistical analysis results supporting Subsection S2.3 (PDF)
