# Supplementary material for: Insights into Aquatic Safety and Environmental Risks of Doped LLZO Solid-State Electrolytes
Source: ACS Omega. 2026 Feb 3;11(6):9290–302. doi: 10.1021/acsomega.5c08890 (PMC12917720; doi:10.1021/acsomega.5c08890)
Supplement: Supplementary file 1 [file ao5c08890_si_001.pdf]

Supporting Information

Insights into Aquatic Safety and Environmental Risks of Doped LLZO

Raphael Martinez Garcia [a, b, +], Marlon Muniz da Silva [c, d, +], Gabriela Helena da Silva [a], Aline Maria Zigiotto de Medeiros [a], Joice Janeri Gomes [a], Diego Stéfani Teodoro Martinez [a,e *], Mathias Strauss [a]

[a] Brazilian Nanotechnology National Laboratory (LNNano), Brazilian Center for Research in Energy and Materials (CNPEM), Street Giuseppe Maximo Scolfaro 10000, 13083-100, Campinas, São Paulo, Brazil.

[b] School of Chemical Engineering (FEQ), University of Campinas (UNICAMP), Avenue Albert Einstein 500, 13083-852, Campinas, São Paulo, Brazil.

[c] Brazilian Synchrotron Light Laboratory (LNLS), Brazilian Center for Research in Energy and Materials (CNPEM), Street Giuseppe Maximo Scolfaro 10000, 13083-100, Campinas, São Paulo, Brazil.

[d] Institute of Chemistry (IQ), University of Campinas (UNICAMP), Street Monteiro Lobato 270, 13083-970, Campinas, São Paulo, Brazil.

[e] School of Technology (FT), University of Campinas (UNICAMP), Street Paschoal Marmo, 1888, 13484-332, Limeira, São Paulo, Brazil.

[+] These authors contributed equally

[*] Corresponding author

Dr. Diego Stéfani Teodoro Martinez (diego.martinez@lnnano.cnpem.br)

S1. Solid-state synthesis of LLZO

The La_2O_3 precursor was calcined at 900 °C for 12 h to eliminate residues of $La(OH)_3$ and LaOOH formed from the interaction La_2O_3 and atmospheric moisture. Stoichiometric quantities of the precursors were used, following the respective nominal formulas of $Li_{6,1}La_3Zr_2Al_{0,3}O_{12}$ for LLZAO and $Li_{6,6}La_3Zr_{1,6}Ta_{0,4}O_{12}$ for LLZTO, except for the Li precursor, which was added in an excess of 15 % wt. to compensate for Li losses by volatilization during calcination. The precursors were measured to produce 6.0 g of both LLZAO and LLZTO.

The precursors were then pulverized and mixed via milling in a high-performance ball mill E_{max} (Retsch) using a 50 mL yttrium-stabilized zirconia (YSZ) vessel with 5 mm diameter YSZ spheres and 10 mL of isopropyl alcohol. Milling was performed at 400 rpm for 6 h, alternating the direction of rotation (clockwise to counterclockwise) every 30 min with a one-minute pause between the inversions. The resulting dispersion was removed from the vessel and dried in an oven overnight.

The dried material was then uniaxially pelletized using a stainless-steel die with a diameter of 15,8 mm under 10 tons of pressure maintained for 10 min. Afterwards, the pellets were calcined at 925 °C for 12 h (5 °C/min) inside a closed alumina crucible. The calcined pellets were then crushed into a fine powder using an agate mortar. A fraction of the calcined powders was separated for characterization, whereas the rest was milled in the high-performance ball mill Emax (Retsch) at 1000 rpm for 6 h, using the same vessel and spheres as before, in 7.5 mL of isopropyl alcohol per gram of material. Afterwards, the samples were dried in an oven overnight and crushed into a fine powder using an agate mortar.

S2. Complementary Results and Discussion

S2.1. Compositional analysis

The X-ray diffractograms of the LLZAO and LLZTO powders (Figure S1A) reveal the presence of Li_2CO_3 and traces of Li_2ZrO_3, $LaAlO_3$ and unidentified secondary phases. The presence of Li_2CO_3 can be attributed to the protonation of LLZO, whereas the other phases are commonly formed during LLZO synthesis. Furthermore, Al-rich regions were identified in the LLZAO sample by SEM–EDS mapping (Figure S1B), whose formation in the solid-state route is attributed to the compositional inhomogeneity inherent to this synthesis method. These regions are likely associated with the formation of $LaAlO_3$ in LLZAO, while other impurities may also have originated from compositional inhomogeneity [1,2].

Comparing the ICP–OES analyses (Figure S1C) of the powders redispersed in UPW after recovery from UPW and RW reveals that contamination of the milled LLZAO and LLZTO powders with metallic species present in the RW (Na, K, Mg and Ca) occurred after exposure to this medium. The concentration values should be interpreted qualitatively rather than quantitatively, as the mass of recovered powders prior to redispersion was not measured. Nevertheless, Na, K, Mg and Ca contamination of the powders exposed to RW likely lead to the formation of carbonates through interaction with atmospheric CO_2 during the drying of the powders [3,4], which may explain the carbonate bands observed in the Raman spectra of the powders after exposure to RW (Figure 1D).

Moreover, Raman spectroscopy of the powders exposed to UPW (30 min sonication, 96 h aging) also revealed residual carbonate features (Figure S1D). Since UPW does not contain significant amounts of Na, K, Mg, or Ca, this carbonate signal is likely associated with traces of Li_2CO_3 originating from unwashed Li ions remaining in the solution. Hence, at least part of the weak carbonate signal detected in the powders after exposure to RW likely arises from Li_2CO_3 residues. Furthermore, some of the counter-anions of the metallic species present in RW (bicarbonate, sulfate, and chloride) likely remained in the powders, as they were not washed after removal from RW. These anions may have formed trace secondary phases upon drying by combining with the metallic species in RW. Finally, as discussed in subsection S2.2, there remains a possibility that these metallic species were incorporated into the LLZO crystal structure rather than forming carbonates upon drying.

S2.2. ICP-OES results and pH behavior

The Li, La, Zr, Al, and Ta concentrations in RW and UPW after exposure to the powders, measured by ICP–OES, were compared both between themselves and against the corresponding blanks (RW and UPW prior to exposure) using a one-way ANOVA with Tukey's HSD post-hoc test. Sonication time and exposure duration were not considered in this analysis. Differences were regarded as significant for p-values < 0.01, and negative concentration values were treated as below the calibration limits of the analysis. According to this procedure (Table S2), for both UPW and RW, only Li and Al (the latter for LLZAO exposure only) concentrations showed significant variations after exposure to the powders. Furthermore, for both media, Li concentration did not differ significantly between LLZAO and LLZTO exposures. However, Li concentration varied significantly between UPW and RW, being notably higher in the latter. In contrast, Al concentration did not differ significantly between UPW and RW.

Linear fits were then employed to investigate whether the release of these elements was influenced by sonication and/or exposure time (Figures S2A–D). For both UPW and RW, Al release from LLZAO appeared to be unaffected by either parameter, as indicated by the very low adjusted R^2 values (< 0.05, Tables S5 and S6). Li release also did not appear to be influenced by sonication or exposure time in RW (adjusted $R^2 < 0.25$, Tables S3 and S4). In contrast, for UPW, there was weak-to-moderate evidence of Li release increasing with exposure time (adjusted $R^2 > 0.25$, Table S3) and strong evidence of Li release increasing with sonication time (adjusted $R^2 > 0.75$, Table S4). Specifically, in UPW, variations in Li concentration were negligible within the first 24 h but became significant at 96 h. Moreover, the Li concentration after 96 h without sonication was comparable to that observed up to 24 h with sonication.

Similarly, pH measurements (Figure S2E) were compared based on their average values and associated measurement errors, which were larger than the corresponding standard deviations. For both UPW and RW, the pH did not vary significantly between LLZAO and LLZTO exposures, although pH values differed significantly between UPW and RW. Linear fits of the pH values over time for each condition revealed strong evidence (adjusted $R^2 > 0.75$, Table S7) of an increase in pH over time in UPW and a slight decrease over time in RW. More specifically, in all cases, pH variations were negligible within the first 24 h but became significant at 96 h. Comparing the pH values after 96 h of exposure in the presence (10 min) and absence of sonication showed that

sonication did not significantly affect the pH in RW. In contrast, in UPW, the pH after 96 h of exposure with sonication was significantly higher than without sonication, the latter remaining within the range of values measured up to 24 h in the presence of sonication.

The Li release initially occurred due to the dissolution of Li_2CO_3, which is likely fast enough to be considered instantaneous relative to the time scales used in this study. In RW, the absence of evidence for an increase in Li concentration or pH over time suggests that no further protonation took place, consistent with the lack of variation in lattice parameters determined by Rietveld refinement (Figure 1C). In contrast, in UPW, the possible increase in Li concentration and the clear rise in pH indicate that LLZO underwent a slight degree of protonation during the final 72 h of exposure.

The comparisons across sonication times reinforce these hypotheses. In RW, sonication did not affect Li release or pH because this medium promotes particle agglomeration, preventing effective deagglomeration and consequently reducing the total surface area. In contrast, sonication induces particle deagglomeration in UPW, increasing the total surface area and facilitating protonation, as this process is governed by the diffusion of H^+ ions within the LLZO structure. Once the particle surface becomes protonated, the hydrogen-rich outer layer limits further protonation of the particle interior [5,6]. At ambient temperature, protonation requires several hours to penetrate beyond 1 μm [7], so large particles and agglomerates of freshly exposed LLZO exhibit lower Li release rates and slower pH increases. Therefore, in UPW, both Li concentration and pH rose significantly only during the last 72 h when sonication was initially applied, and not in its absence.

Protonation may also have been favored in UPW because it is more acidic [5] than RW, a difference that likely arises from their exposure to air during sample preparation. Under such conditions, UPW is prone to slight acidification by atmospheric CO_2 within a few seconds [8–10], decreasing its pH to approximately 5 ± 1 (typically around 5.7 [9]) prior to LLZO exposure. In contrast, RW contains dissolved salts that buffer the pH against CO_2-induced acidification and significantly slow down this process [9,10], thereby maintaining the pH at 6.7 ± 0.3 before LLZO exposure. For RW, the slight decrease in pH observed during the last 72 h of exposure to LLZO may thus be attributed to gradual CO_2-induced acidification.

The higher Li concentration in RW compared with UPW could possibly be explained by the substitution of Li^+ ions in the protonated LLZO crystal structure by Na^+, K^+, Mg^{2+} and/or Ca^{2+}

ions from RW, a process that may have been facilitated by the prior increase in lattice parameters due to protonation during milling. While this hypothesis is consistent with the detection of these elements by ICP–OES in the powders after exposure to RW (Figure S1C), such substitution often alters lattice parameters because of differences in ionic radii between Li^+ and the doping ions [11–13] and can significantly affect the Raman spectra [12,14–17]. Therefore, the absence of lattice parameter changes after exposure to RW, as determined by Rietveld refinement (Figure 1C), together with the lack of significant differences in the Raman spectra (apart from the reduction in the Li_2CO_3 signal) after exposure to RW (Figure 1D), suggests that this explanation is unlikely. The most likely reason for the higher Li concentration in RW is that it may have been overestimated, as the metallic species added to RW (Na, K, Mg, and Ca) can interfere with Li measurements by ICP–OES [18].

Assuming that the blank-subtracted Li concentration measured in the UPW samples exposed to LLZO for 15 minutes arises solely from Li_2CO_3 dissolution, it was possible to calculate the mass fraction f of Li_2CO_3 in the milled powders prior to immersion in the aquatic media, according to Equation S1:

$$f = \frac{\left(\frac{[Li]}{2}\right) M_{Li_2CO_3}}{[Powder]_0} \quad \text{(S1)}$$

In this expression $[Li]$ represents the blank-subtracted molar concentration of Li in the solution in L^{-1}, M is the molar mass in mol g^{-1} and $[Powder]_0$ is the concentration of powder added to the aquatic media in g L^{-1}, which was nominally 0.1 g L^{-1}. Considering the stoichiometries defined in Equations 1 and 2, the degree of protonation was then calculated using Equations S2 through S4:

$$f = \frac{\left(\frac{x}{2}\right) M_{Li_2CO_3}}{M_{LLZO} + \left(\frac{x}{2}\right) M_{Li_2CO_3}} \quad \text{(S2)}$$

$$M_{LLZO} = (7 - 3a - b - x)M_{Li} + xM_H + aM_{Al} + 3M_{La} + (2 - b)M_{Zr} + bM_{Ta} + 12M_O \quad \text{(S3)}$$

$$P = \frac{x}{7 - 3a - b} \quad \text{(S4)}$$

In this expression x, a and b represent the stoichiometries of H, Al, and Ta in the milled powder, and P is the degree of protonation, defined as the fraction of Li lost from LLZO due to protonation.

Considering the nominal Al and Ta stoichiometries of LLZAO and LLZTO, these calculations yielded P values of 61 ± 3 % for LLZAO and 60 ± 3 % for LLZTO.

Similarly, the fraction A of Al leached from LLZAO upon immersion in UPW or RW was calculated by dividing the blank-subtracted Al concentration by the total moles of Al in the LLZO fraction of the milled powder, according to Equation S5:

$$A = \frac{[Al]}{a(1-f)[Powder]_0/M_{LLZO}} \quad \text{(S5)}$$

Here, $[Al]$ represents the blank-subtracted molar concentration of Al in the solution in mol L^{-1}. Since Al release was independent of the aqueous medium, sonication, and exposure time, the average $[Al]$ value from all LLZAO exposure conditions was used, yielding an A value of 8.7 ± 1.5 %.

S2.3. FET assay statistical analysis

The FET assay data were tested for normality using the Kolmogorov–Smirnov test and for homogeneity of variance using Levene's test. When these assumptions were satisfied, a two-way ANOVA followed by Tukey's post hoc test was performed to compare the data. Differences were considered statistically significant at $p < 0.05$. According to this analysis (Table S8), no statistically significant differences in zebrafish length were observed between any of the exposure conditions and the control groups.

References

(1) Wang, Y.; Chen, Z.; Jiang, K.; Shen, Z.; Passerini, S.; Chen, M. Accelerating the Development of LLZO in Solid-State Batteries Toward Commercialization: A Comprehensive Review. *Small* **2024**, *20* (35), 2402035. https://doi.org/10.1002/smll.202402035.

(2) Parascos, K.; Watts, J. L.; Alarco, J. A.; Chen, Y.; Talbot, P. C. Compositional and Structural Control in LLZO Solid Electrolytes. *RSC Advances* **2022**, *12* (36), 23466–23480. https://doi.org/10.1039/d2ra03303h.

(3) Santos, H. S.; Nguyen, H.; Venâncio, F.; Ramteke, D.; Zevenhoven, R.; Kinnunen, P. Mechanisms of Mg Carbonates Precipitation and Implications for CO_2 Capture and

Utilization/Storage. *Inorganic Chemistry Frontiers* **2023**, *10* (9), 2507–2546. https://doi.org/10.1039/d2qi02482a.

(4) Lamaa, G.; Duarte, A. P. C.; Silva, R. V.; De Brito, J. Carbonation of Alkali-Activated Materials: A Review. *Materials* **2023**, *16* (8), 3086. https://doi.org/10.3390/ma16083086.

(5) Redhammer, G. J.; Badami, P.; Meven, M.; Ganschow, S.; Berendts, S.; Tippelt, G.; Rettenwander, D. Wet-Environment-Induced Structural Alterations in Single- And Polycrystalline LLZTO Solid Electrolytes Studied by Diffraction Techniques. *ACS Applied Materials and Interfaces* **2021**, *13* (1), 350–359. https://doi.org/10.1021/acsami.0c16016.

(6) Ye, R.; Ihrig, M.; Imanishi, N.; Finsterbusch, M.; Figgemeier, E. A Review on Li^+/H^+ Exchange in Garnet Solid Electrolytes: From Instability against Humidity to Sustainable Processing in Water. *ChemSusChem* **2021**, *14* (20), 4397–4407. https://doi.org/10.1002/cssc.202101178.

(7) Hiebl, C.; Young, D.; Wagner, R.; Wilkening, H. M. R.; Redhammer, G. J.; Rettenwander, D. Proton Bulk Diffusion in Cubic $Li_7La_3Zr_2O_{12}$ Garnets as Probed by Single X-Ray Diffraction. *The Journal of Physical Chemistry C* **2019**, *123* (2), 1094–1098. https://doi.org/10.1021/acs.jpcc.8b10694.

(8) Talling, J. F. pH, the CO_2 System and Freshwater Science. *Freshwater Reviews* **2010**, *3* (2), 133–146. https://doi.org/10.1608/frj-3.2.156.

(9) Lower, S. K. Carbonate Equilibria in Natural Waters, 1999.

(10) Dreybrodt, W.; Lauckner, J.; Zaihua, L.; Svensson, U.; Buhmann, D. The Kinetics of the Reaction $CO_2 + H_2O \rightarrow H^+ + HCO_3^-$ as One of the Rate Limiting Steps for the Dissolution of Calcite in the System H_2O-CO_2-$CaCO_3$. *Geochimica et Cosmochimica Acta* **1996**, *60* (18), 3375–3381. https://doi.org/10.1016/0016-7037(96)00181-0.

(11) Hu, Z.; Liu, H.; Ruan, H.; Hu, R.; Su, Y.; Zhang, L. High Li-Ion Conductivity of Al-Doped $Li_7La_3Zr_2O_{12}$ Synthesized by Solid-State Reaction. *Ceramics International* **2016**, *42* (10), 12156–12160. https://doi.org/10.1016/j.ceramint.2016.04.149.

(12) Meesala, Y.; Liao, Y. K.; Jena, A.; Yang, N. H.; Pang, W. K.; Hu, S. F.; Chang, H.; Liu, C. E.; Liao, S. C.; Chen, J. M.; Guo, X.; Liu, R. S. An Efficient Multi-Doping Strategy to Enhance Li-Ion Conductivity in the Garnet-Type Solid Electrolyte $Li_7La_3Zr_2O_{12}$. *Journal of Materials Chemistry A* **2019**, *7* (14), 8589–8601. https://doi.org/10.1039/c9ta00417c.

(13) Amardeep; Kobi, S.; Mukhopadhyay, A. Mg-Doping towards Enhancing the Composition-Phase-Structural Stability of Li-La-Zirconate Based Cubic Garnet upon Exposure to Air. *Scripta Materialia* **2019**, *162*, 214–218. https://doi.org/10.1016/j.scriptamat.2018.11.026.

(14) Thompson, T.; Wolfenstine, J.; Allen, J. L.; Johannes, M.; Huq, A.; David, I. N.; Sakamoto, J. Tetragonal vs. Cubic Phase Stability in Al – Free Ta Doped $Li_7La_3Zr_2O_{12}$ (LLZO). *Journal of Materials Chemistry A* **2014**, *2* (33), 13431–13436. https://doi.org/10.1039/C4TA02099E.

(15) Tietz, F.; Wegener, T.; Gerhards, M. T.; Giarola, M.; Mariotto, G. Synthesis and Raman Micro-Spectroscopy Investigation of $Li_7La_3Zr_2O_{12}$. *Solid State Ionics* **2013**, *230*, 77–82. https://doi.org/10.1016/j.ssi.2012.10.021.

(16) Larraz, G.; Orera, A.; Sanjuán, M. L. Cubic Phases of Garnet-Type $Li_7La_3Zr_2O_{12}$: The Role of Hydration. *Journal of Materials Chemistry A* **2013**, *1* (37), 11419–11419. https://doi.org/10.1039/c3ta11996c.

(17) Košir, J.; Mousavihashemi, S.; Suominen, M.; Kobets, A.; Wilson, B. P.; Rautama, E.-L.; Kallio, T. Supervalent Doping and Its Effect on the Thermal, Structural and Electrochemical Properties of $Li_7La_3Zr_2O_{12}$ Solid Electrolytes. *Materials Advances* **2024**, *5* (12), 5260–5274. https://doi.org/10.1039/d4ma00119b.

(18) Han, T.; Yu, X.; Guo, Y.; Li, M.; Deng, T. Determination of Lithium in High Salinity Samples by Inductively Coupled Plasma Optical Emission Spectrometry (ICP-OES). *Spectroscopy and Spectral Analysis* **2020**, *40* (4), 1214. http://dx.doi.org/10.3964/j.issn.1000-0593(2020)04-1214-07.

S3. Tables

Table S1. Physicochemical characteristics of the milled LLZAO and LLZTO powders. Particle size and polydispersity index (PDI) were determined by DLS after 90 min of sonication.

Powder	Average Size (nm)*	Polydispersity Index	Surface Area ($m^2\ g^{-1}$)	Zeta Potential (mV)
**Milled LLZTO**	254 ± 6	0.20 ± 0.01	16.03 ± 0.07	-40 ± 3
**Milled LLZAO**	262 ± 4	0.15 ± 0.04	15.87 ± 0.06	-39 ± 3

** Average hydrodynamic diameter.*

Table S2 Statistical analysis of Li, La, Zr, Al, and Ta concentrations between different pairs of exposure conditions (each condition represented by dispersed powder, if present, followed by the aqueous medium), as determined by one-way ANOVA. Values correspond to p-values determined by Tukey's HSD post-hoc analysis. Grey cells correspond to statistically significant differences (p-value < 0.01).

Pair Comparison	[Li]	[La]	[Zr]	[Al]	[Ta]
**UPW - RW**	1.000	1.000	0.924	0.995	1.000
**LLZTO UPW - LLZTO RW**	0.000	0.034	0.068	0.001	0.019
**LLZAO UPW - LLZAO RW**	0.000	0.751	0.793	0.175	0.784
**LLZTO UPW - LLZAO UPW**	0.057	1.000	0.001	0.000	0.000
**LLZTO RW - LLZAO RW**	0.527	0.578	1.000	0.000	0.973
**LLZTO UPW - UPW**	0.000	1.000	0.138	0.057	0.069
**LLZAO UPW - UPW**	0.000	1.000	1.000	0.000	1.000
**LLZTO RW - RW**	0.000	0.568	1.000	1.000	0.832
**LLZAO RW - RW**	0.000	0.986	1.000	0.000	0.974

Table S3. Linear fit (Figure S2A) parameters for the concentration of Li (blank-subtracted) in RW and UPW exposed to 100 mg L^{-1} of milled LLZAO and LLZTO as a function of exposure time (sonication for 10 min).

**Powder**	**Milled LLZAO**		**Milled LLZTO**	
**Medium**	**UPW**	**RW**	**UPW**	**RW**
**Intercept**	376	519	408	534
**Slope**	1.14	0.54	0.74	0.38
**adj. R^2**	0.69	0.18	0.42	0.12

Table S4. Linear fit (Figure S2B) parameters for the concentration of Li (blank-subtracted) in RW and UPW exposed to 100 mg L^{-1} of milled LLZAO and LLZTO for 96 h as a function of sonication time.

**Powder**	**Milled LLZAO**		**Milled LLZTO**	
**Medium**	**UPW**	**RW**	**UPW**	**RW**
**Intercept**	364	590	387	590
**Slope**	11.64	-1.93	7.33	-1.20
**adj. R^2**	0.98	-0.03	0.81	-0.19

Table S5. Linear fit (Figure S2C) parameters for the concentration of Al (blank-subtracted) in RW and UPW exposed to 100 mg L^{-1} of milled LLZAO as a function of exposure time (sonication for 10 min).

**Medium**	**UPW**	**RW**
**Intercept**	3.22	2.68
**Slope**	-0.003	-0.003
**adj. R^2**	-0.02	0.00

Table S6. Linear fit (Figure S2D) parameters for the concentration of Al (blank-subtracted) in RW and UPW exposed to 100 mg L^{-1} of milled LLZAO for 96 h as a function of sonication time.

**Case**	**UPW**	**RW**
**Intercept**	2.69	1.98
**Slope**	-0.004	0.016
**adj. R^2**	-0.25	-0.21

Table S7. Linear fit (Figure S2E) parameters for the pH in RW and UPW exposed to 100 mg L^{-1} of milled LLZAO and LLZTO as a function of exposure time (sonication for 10 min).

**Powder**	**Milled LLZAO**		**Milled LLZTO**	
**Medium**	**UPW**	**RW**	**UPW**	**RW**
**Intercept**	6.8	7.9	6.8	8.2
**Slope**	0.014	-0.006	0.011	-0.009
**adj. R^2**	0.81	0.93	0.89	0.77

Table S8. Statistical analysis of the FET zebrafish assay, as determined through two-way Anova. Values correspond to p-values determined by Tukey HSD post-hoc analysis. Note that all p-values are higher than 0.05.

**With chorium**		**Without chorium**	
**Comparison**	**p-value**	**Comparison**	**p-value**

LLZAO 1 mg L^{-1} - LLZAO 0 mg L^{-1}	1.000	LLZAO 1 mg L^{-1} - LLZAO 0 mg L^{-1}	0.976
LLZAO 10 mg L^{-1} - LLZAO 0 mg L^{-1}	0.996	LLZAO 10 mg L^{-1} - LLZAO 0 mg L^{-1}	0.822
LLZAO 10 mg L^{-1} - LLZAO 1 mg L^{-1}	1.000	LLZAO 10 mg L^{-1} - LLZAO 1 mg L^{-1}	1.000
LLZAO 100 mg L^{-1} - LLZAO 0 mg L^{-1}	0.998	LLZAO 100 mg L^{-1} - LLZAO 0 mg L^{-1}	0.919
LLZAO 100 mg L^{-1} - LLZAO 1 mg L^{-1}	0.936	LLZAO 100 mg L^{-1} - LLZAO 1 mg L^{-1}	1.000
LLZAO 100 mg L^{-1} - LLZAO 10 mg L^{-1}	0.862	LLZAO 100 mg L^{-1} - LLZAO 10 mg L^{-1}	1.000
LLZTO 0 mg L^{-1} - LLZAO 0 mg L^{-1}	1.000	LLZTO 0 mg L^{-1} - LLZAO 0 mg L^{-1}	1.000
LLZTO 0 mg L^{-1} - LLZAO 1 mg L^{-1}	1.000	LLZTO 0 mg L^{-1} - LLZAO 1 mg L^{-1}	0.976
LLZTO 0 mg L^{-1} - LLZAO 10 mg L^{-1}	0.996	LLZTO 0 mg L^{-1} - LLZAO 10 mg L^{-1}	0.822
LLZTO 0 mg L^{-1} - LLZAO 100 mg L^{-1}	0.998	LLZTO 0 mg L^{-1} - LLZAO 100 mg L^{-1}	0.919
LLZTO 1 mg L^{-1} - LLZAO 0 mg L^{-1}	0.999	LLZTO 1 mg L^{-1} - LLZAO 0 mg L^{-1}	0.576
LLZTO 1 mg L^{-1} - LLZAO 1 mg L^{-1}	0.965	LLZTO 1 mg L^{-1} - LLZAO 1 mg L^{-1}	0.987
LLZTO 1 mg L^{-1} - LLZAO 10 mg L^{-1}	0.912	LLZTO 1 mg L^{-1} - LLZAO 10 mg L^{-1}	1.000
LLZTO 1 mg L^{-1} - LLZAO 100 mg L^{-1}	1.000	LLZTO 1 mg L^{-1} - LLZAO 100 mg L^{-1}	0.998
LLZTO 1 mg L^{-1} - LLZTO 0 mg L^{-1}	0.999	LLZTO 1 mg L^{-1} - LLZTO 0 mg L^{-1}	0.576
LLZTO 10 mg L^{-1} - LLZAO 0 mg L^{-1}	0.848	LLZTO 10 mg L^{-1} - LLZAO 0 mg L^{-1}	0.858
LLZTO 10 mg L^{-1} - LLZAO 1 mg L^{-1}	0.986	LLZTO 10 mg L^{-1} - LLZAO 1 mg L^{-1}	1.000
LLZTO 10 mg L^{-1} - LLZAO 10 mg L^{-1}	0.997	LLZTO 10 mg L^{-1} - LLZAO 10 mg L^{-1}	1.000
LLZTO 10 mg L^{-1} - LLZAO 100 mg L^{-1}	0.436	LLZTO 10 mg L^{-1} - LLZAO 100 mg L^{-1}	1.000
LLZTO 10 mg L^{-1} - LLZTO 0 mg L^{-1}	0.848	LLZTO 10 mg L^{-1} - LLZTO 0 mg L^{-1}	0.858
LLZTO 10 mg L^{-1} - LLZTO 1 mg L^{-1}	0.520	LLZTO 10 mg L^{-1} - LLZTO 1 mg L^{-1}	1.000
LLZTO 100 mg L^{-1} - LLZAO 0 mg L^{-1}	1.000	LLZTO 100 mg L^{-1} - LLZAO 0 mg L^{-1}	0.558
LLZTO 100 mg L^{-1} - LLZAO 1 mg L^{-1}	1.000	LLZTO 100 mg L^{-1} - LLZAO 1 mg L^{-1}	0.986
LLZTO 100 mg L^{-1} - LLZAO 10 mg L^{-1}	0.999	LLZTO 100 mg L^{-1} - LLZAO 10 mg L^{-1}	1.000
LLZTO 100 mg L^{-1} - LLZAO 100 mg L^{-1}	0.990	LLZTO 100 mg L^{-1} - LLZAO 100 mg L^{-1}	0.998
LLZTO 100 mg L^{-1} - LLZTO 0 mg L^{-1}	1.000	LLZTO 100 mg L^{-1} - LLZTO 0 mg L^{-1}	0.558
LLZTO 100 mg L^{-1} - LLZTO 1 mg L^{-1}	0.996	LLZTO 100 mg L^{-1} - LLZTO 1 mg L^{-1}	1.000
LLZTO 100 mg L^{-1} - LLZTO 10 mg L^{-1}	0.923	LLZTO 100 mg L^{-1} - LLZTO 10 mg L^{-1}	1.000

S4. Figures

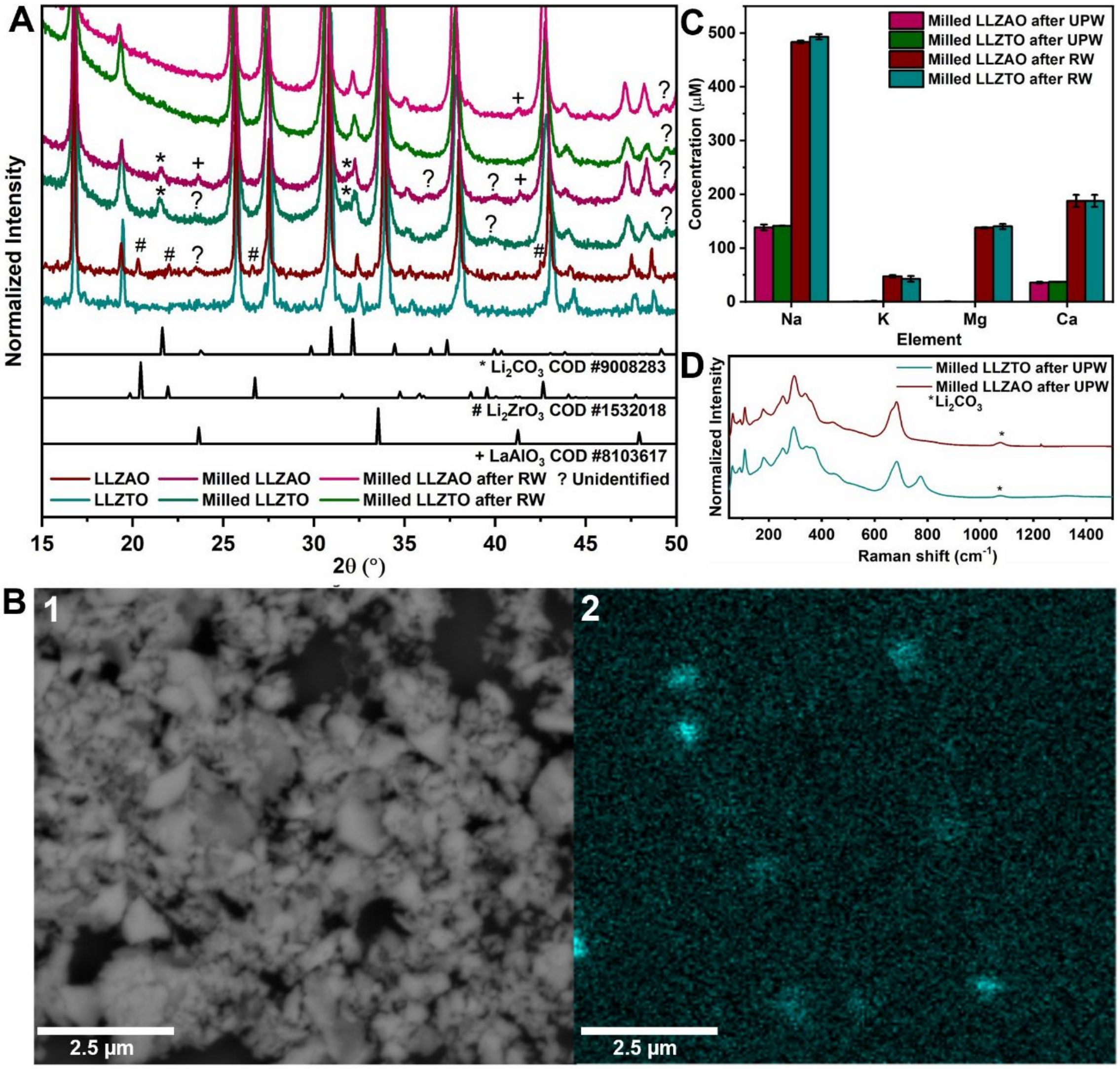

Figure S1. (A) Zoomed-in XRD diffractograms of LLZTO and LLZAO samples before milling, after milling, and after exposure to RW. All unassigned peaks correspond to cubic LLZO. (B) SEM micrographs of milled LLZAO after 96 h exposure to RW with 30 min of sonication: (1) general micrograph and (2) Al distribution measured by EDS (Kα emission) in the same area. (C) Concentrations of Na, K, Mg, and Ca obtained from ICP–OES measurements of redispersions, in UPW, of milled LLZAO and LLZTO powders previously aged for 96 h with 30 min of sonication in UPW and RW. Error bars indicate standard deviation; values are provided for qualitative interpretation. (D) Raman spectra of milled LLZTO and LLZAO samples after 96 h exposure to UPW with 30 min of sonication.

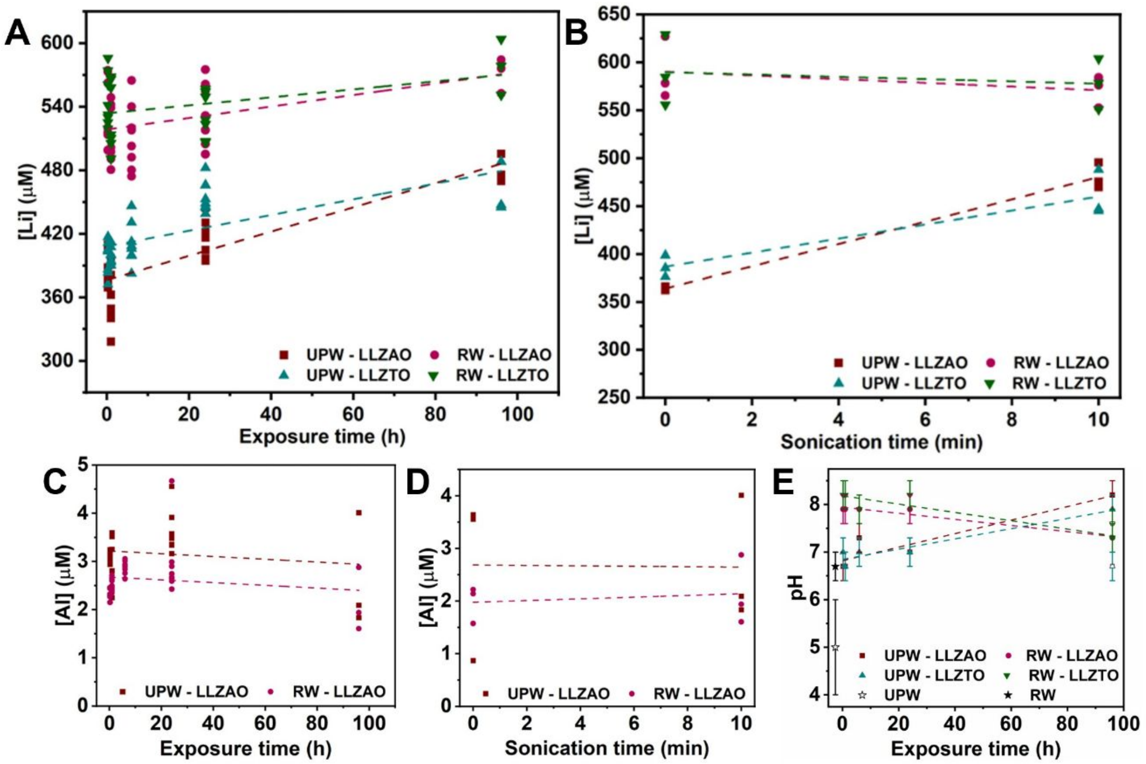

Figure S2. (A) Li concentration in RW and UPW exposed to 100 mg L⁻¹ of milled LLZAO and LLZTO as a function of exposure time (10 min sonication). (B) Li concentration in RW and UPW exposed to 100 mg L⁻¹ of milled LLZAO and LLZTO for 96 h as a function of sonication time. (C) Al concentration in RW and UPW exposed to 100 mg L⁻¹ of milled LLZAO as a function of exposure time (10 min sonication). (D) Al concentration in RW and UPW exposed to 100 mg L⁻¹ of milled LLZAO for 96 h as a function of sonication time. All concentrations were determined by ICP–OES; subtraction of the respective blank (UPW and RW) concentrations was applied. (E) pH values in RW and UPW exposed to 100 mg L⁻¹ of milled LLZAO and LLZTO as a function of exposure time (10 min sonication). Points for UPW and RW represent pH values of blanks and are not related to exposure time. Colorless dots at 96 h represent pH values of the exposure conditions after 96 h without sonication. All dashed lines represent linear fits; fitting parameters are provided in Tables S4–S7.
